# Gadd45a Protein Promotes Skeletal Muscle Atrophy by Forming a Complex with the Protein Kinase MEKK4*[Fn FN3]^♦^

**DOI:** 10.1074/jbc.M116.740308

**Published:** 2016-06-29

**Authors:** Steven A. Bullard, Seongjin Seo, Birgit Schilling, Michael C. Dyle, Jason M. Dierdorff, Scott M. Ebert, Austin D. DeLau, Bradford W. Gibson, Christopher M. Adams

**Affiliations:** From the ‡ Department of Internal Medicine,; § Fraternal Order of Eagles Diabetes Research Center, and; Departments of ¶ Molecular Physiology and Biophysics and; ** Ophthalmology and Visual Sciences, University of Iowa, Iowa City, Iowa 52242,; the ‖ Iowa City Veterans Affairs Medical Center, Iowa City, Iowa 52246,; §§ Emmyon, Inc., Coralville, Iowa 52241,; the ‡‡ Buck Institute for Research on Aging, Novato, California 94945, and; the ¶¶ Department of Pharmaceutical Chemistry, University of California at San Francisco, San Francisco, California 94143

**Keywords:** aging, mass spectrometry (MS), muscle, muscle atrophy, skeletal muscle, skeletal muscle metabolism, Gadd45a, MEKK4

## Abstract

Skeletal muscle atrophy is a serious and highly prevalent condition that remains poorly understood at the molecular level. Previous work found that skeletal muscle atrophy involves an increase in skeletal muscle Gadd45a expression, which is necessary and sufficient for skeletal muscle fiber atrophy. However, the direct mechanism by which Gadd45a promotes skeletal muscle atrophy was unknown. To address this question, we biochemically isolated skeletal muscle proteins that associate with Gadd45a as it induces atrophy in mouse skeletal muscle fibers *in vivo*. We found that Gadd45a interacts with multiple proteins in skeletal muscle fibers, including, most prominently, MEKK4, a mitogen-activated protein kinase kinase kinase that was not previously known to play a role in skeletal muscle atrophy. Furthermore, we found that, by forming a complex with MEKK4 in skeletal muscle fibers, Gadd45a increases MEKK4 protein kinase activity, which is both sufficient to induce skeletal muscle fiber atrophy and required for Gadd45a-mediated skeletal muscle fiber atrophy. Together, these results identify a direct biochemical mechanism by which Gadd45a induces skeletal muscle atrophy and provide new insight into the way that skeletal muscle atrophy occurs at the molecular level.

## Introduction

Skeletal muscle wasting, or atrophy, occurs in many people as they become older, experience muscle disuse, and/or suffer from malnutrition, critical illness, or advanced chronic illness. At the cellular level, skeletal muscle atrophy is explained by a reduction in the size of skeletal muscle fibers. At the molecular level, skeletal muscle atrophy is highly complex, poorly understood, and still largely unexplored.

Over the past several years, a few proteins have emerged as important mediators of skeletal muscle atrophy, and one of these proteins is called growth arrest and DNA damage-inducible 45α or Gadd45a (1). Gadd45a is a soluble, primarily nuclear 18-kDa protein with no known enzymatic activity (2, 3). Under basal conditions, skeletal muscle Gadd45a expression is very low. However, a variety of stress conditions that cause skeletal muscle atrophy (*i.e.* aging, muscle disuse, starvation, and severe illness) activate certain transcription regulators within skeletal muscle fibers (ATF4, HDAC4, and/or FoxOs, depending on the stress) that strongly induce the *Gadd45a* gene (1, 4–17).

Importantly, the induction of skeletal muscle Gadd45a expression is required for skeletal muscle fiber atrophy during at least some stress conditions (*e.g.* starvation, limb immobilization, and muscle denervation (1)). Furthermore, forced expression of Gadd45a is sufficient to induce skeletal muscle fiber atrophy in the absence of an upstream stress (1). Thus, previous findings demonstrated a key role for Gadd45a in skeletal muscle atrophy. However, the direct mechanism by which Gadd45a causes muscle atrophy is not known. This represents an important gap in our current understanding of skeletal muscle atrophy.

In addition to its role in skeletal muscle atrophy, Gadd45a mediates stress responses in other cell types (2, 3). Similar to the situation in skeletal muscle, Gadd45a expression is typically very low when cells are healthy, but it rises dramatically when cells are exposed to a variety of stress conditions. In non-muscle cells, Gadd45a controls stress responses by directly interacting with other proteins to modify their function. Depending on the specific context (cell type, stress, etc.), Gadd45a may bind and regulate certain protein kinases, nucleotide excision repair proteins, base excision repair proteins, nuclear hormone receptors, and/or cell cycle regulators (2, 3, 18). These interactions between Gadd45a and its target proteins lead to context-specific cellular responses, which may include growth inhibition, cell cycle arrest, DNA damage repair, active DNA demethylation, apoptosis, or cellular senescence.

Proteins that interact with Gadd45a in skeletal muscle have not been identified. However, drawing upon findings from other cell types, we hypothesized that Gadd45a might cause skeletal muscle atrophy by directly binding and regulating another protein in skeletal muscle fibers. To test this hypothesis, we conducted an unbiased search for proteins that interact with Gadd45a in mouse skeletal muscle fibers *in vivo*.

## Results

### 

#### 

##### Identification of Proteins That Interact with Gadd45a during Skeletal Muscle Atrophy

**FIGURE 1.**
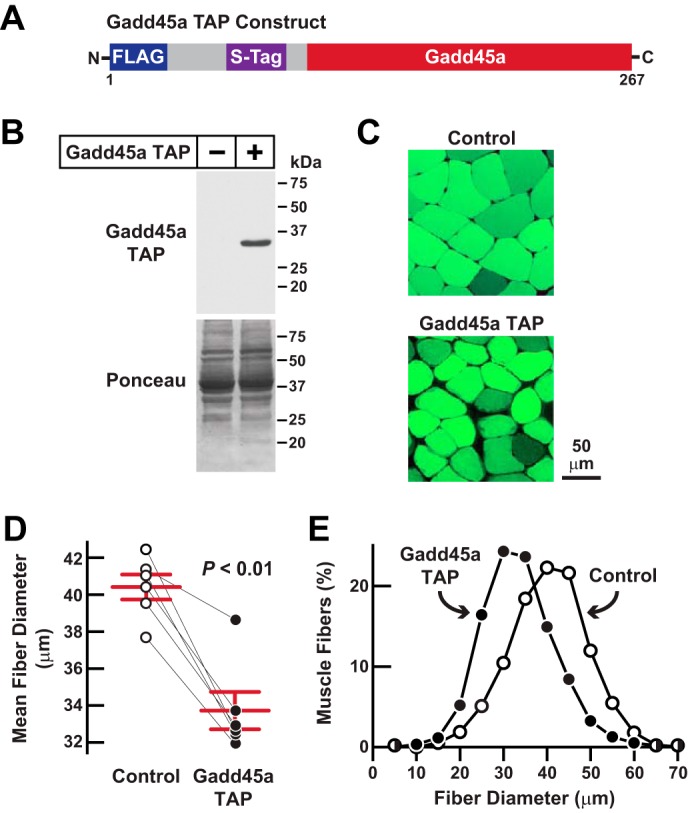
**Gadd45a TAP, a functional Gadd45a construct designed for tandem affinity purification in mouse skeletal muscle.** *A,* schematic of the Gadd45a TAP construct. *B–E,* mouse TA muscle fibers were transfected with 20 μg of Gadd45a TAP plasmid plus 2.5 μg of eGFP plasmid. In each mouse, the contralateral TA muscle fibers (*Control*) were transfected with 20 μg of empty TAP plasmid plus 2.5 μg of eGFP plasmid. Bilateral TA muscles were harvested for analysis 7 days post-transfection. *B,* skeletal muscle protein extracts were subjected to immunoblot analysis using monoclonal anti-FLAG IgG. *C,* representative fluorescence microscopy images of muscle cross-sections. *D,* average diameters of skeletal muscle fibers. Each data point represents the mean of >450 muscle fibers from one muscle, and *horizontal bars* denote average of the means ± S.E. *p* value was determined with a paired *t* test. *E,* size distribution of all muscle fibers from *D*.

A previously published NMR analysis of Gadd45a revealed a globular protein with a disordered flexible region at its N terminus (19). To develop a Gadd45a construct suitable for tandem affinity purification (TAP),^2^ we placed two affinity tags (FLAG and S-tag) at the N terminus of Gadd45a, generating a protein that we termed Gadd45a TAP (Fig. 1*A*). To confirm that the affinity tags in Gadd45a TAP did not interfere with Gadd45a-mediated skeletal muscle atrophy, we transfected a plasmid encoding Gadd45a TAP into the tibialis anterior (TA) skeletal muscles of living mice. In each mouse, the contralateral TA muscle was transfected with empty plasmid and served as an intrasubject control. To detect transfected muscle fibers, bilateral TA muscles were co-transfected with plasmid-encoding eGFP, a transfection marker that does not alter skeletal muscle fiber size. It is important to note that *in vivo* electroporation transfects differentiated muscle fibers, but not satellite cells or connective tissue cells (20). Seven days after skeletal muscle fiber transfection, we euthanized the mice and harvested bilateral TA muscles for biochemical and histological analyses. As expected, transfection of the Gadd45a TAP plasmid generated an ≈30-kDa Gadd45a TAP protein in mouse skeletal muscle (Fig. 1*B*). Furthermore, Gadd45a TAP significantly reduced skeletal muscle fiber size (Fig. 1, *C–E*), indicating that Gadd45a TAP induces skeletal muscle fiber atrophy *in vivo*, similar to untagged Gadd45a.

**FIGURE 2.**
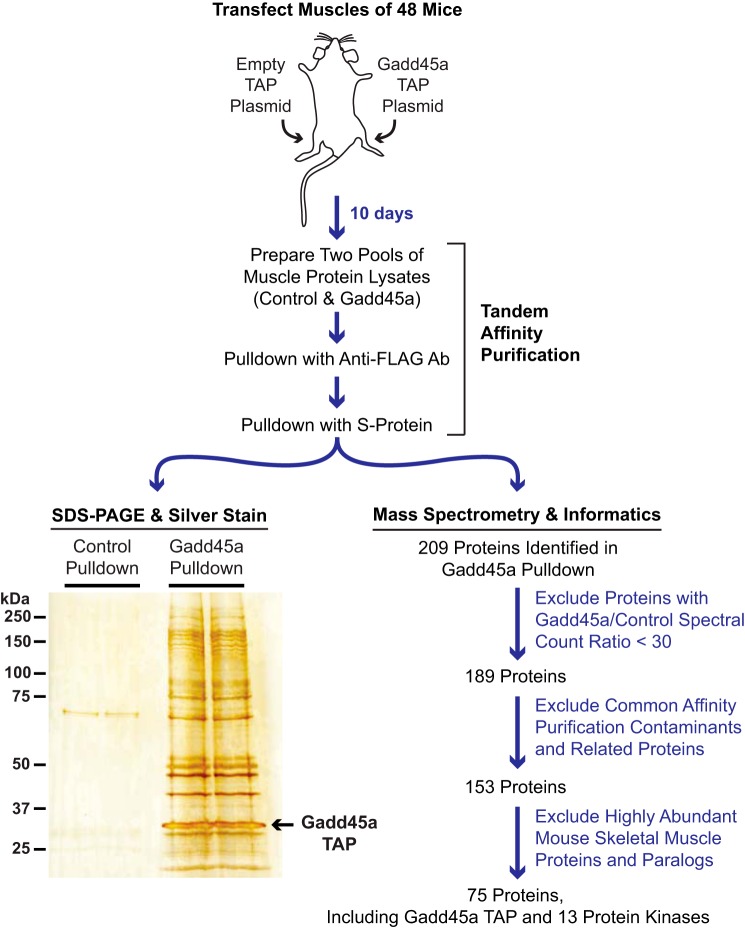
**Isolation and analysis of proteins that interact with Gadd45a TAP in mouse skeletal muscle fibers.** TA skeletal muscle fibers of 48 mice were transfected with 20 μg of empty TAP plasmid (one TA per mouse) or 20 μg of Gadd45a TAP plasmid (the contralateral TA in each mouse). Ten days post-transfection, bilateral TA muscles were harvested and used to prepare pooled protein extracts from each of the two groups of skeletal muscles (control and Gadd45a). The pooled protein extracts were then subjected to sequential purification steps with anti-FLAG magnetic beads and S-protein affinity gel. An aliquot of each final pulldown sample was visualized by SDS-PAGE and silver staining, as shown. The remaining portions of control and Gadd45a pulldown samples were subjected to mass spectrometry and a data analysis workflow that is summarized here and fully detailed under “Experimental Procedures” and the supplemental Tables.

To discover proteins that interact with Gadd45a during skeletal muscle atrophy, we performed a larger scale experiment that is illustrated in Fig. 2. We began by transfecting the TA muscle fibers of 48 mice. In each mouse, one TA was transfected with empty plasmid and the other TA was transfected with Gadd45a TAP plasmid. Ten days after transfection, we euthanized the mice and harvested bilateral TA muscles from each mouse. We then prepared and pooled protein extracts from each of the two groups of skeletal muscles (control and Gadd45a) and subjected both pooled protein extracts to sequential purification steps with anti-FLAG magnetic beads and S-protein-agarose. A small aliquot of each final pulldown sample was visualized by SDS-PAGE and silver staining, and the remaining portions of control and Gadd45a pulldown samples were subjected to mass spectrometric analysis.

**FIGURE 3.**
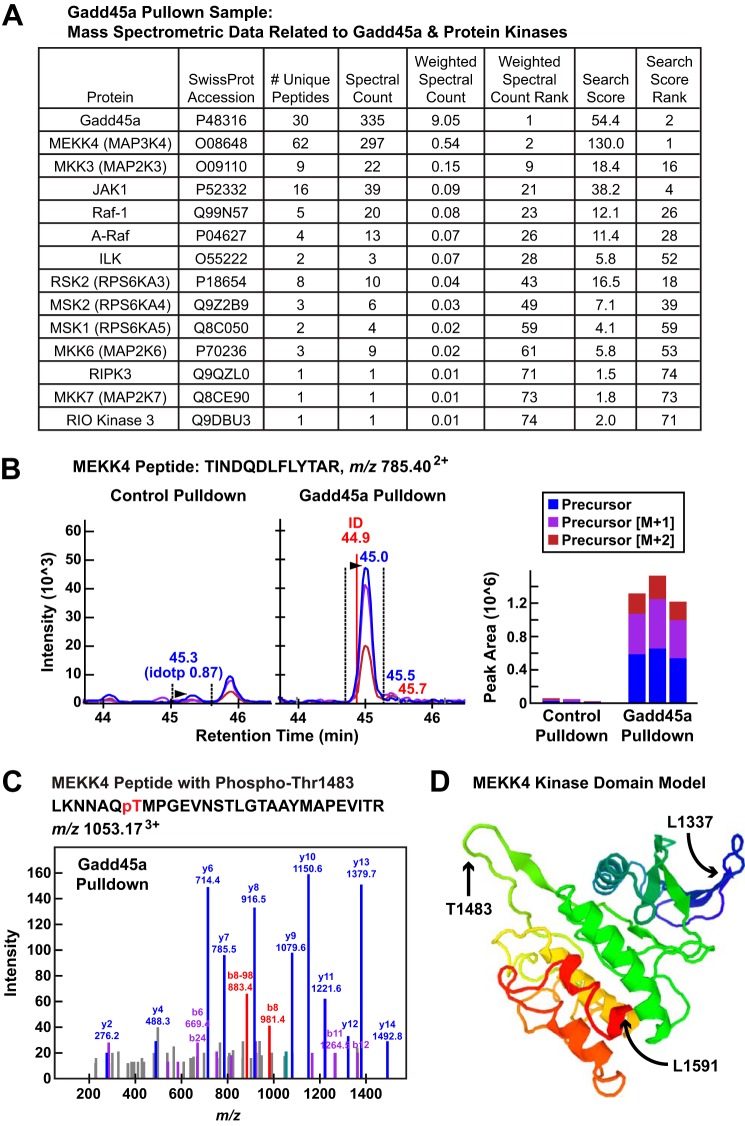
**Gadd45a TAP interacts with multiple protein kinases in mouse skeletal muscle, including MEKK4.** *A,* summary of mass spectroscopic data for Gadd45a and associated protein kinases in the Gadd45a pulldown sample. *B,* data from a representative MEKK4 peptide (TINDQDLFLYTAR with the precursor ion at *m/z* 785.40^2+^) identified in the Gadd45a pulldown sample. *Left* and *middle panels,* quantification using MS1 filtering; the panels show extracted ion chromatograms for the MS1 precursor ions in the control and Gadd45a pulldown samples. *Right panel,* calculated peak areas under the curve for three technical replicates of the control and Gadd45a pulldown samples. *C,* MS/MS of MEKK4 phosphopeptide in the Gadd45a pulldown sample. The b8 ion (*m*/*z* 981.4) and the corresponding b8-98 ion (*m*/*z* 883.4), resulting from a neutral loss of H_3_PO_4_ (−*98*), clearly indicates that phosphorylation in this peptide is located at threonine 1483. *D,* three-dimensional model of the mouse MEKK4 kinase domain (Sprot number O08648, residues 1337–1591), indicating the position of threonine 1483 within the activation loop. The model, generated by The Protein Model Portal, is based on the crystal structure of the Mst1 kinase (template 3comA, sequence identity 33%).

SDS-PAGE and silver staining revealed multiple proteins that were present in the Gadd45a pulldown sample but absent in the control sample (Fig. 2). Using mass spectrometry, we identified 209 proteins in the Gadd45a pulldown sample and 29 proteins in the control sample (supplemental Table 1). To begin to differentiate *bona fide* Gadd45a-interacting proteins from nonspecific interactors, we estimated protein abundance via spectral counting and weighted spectral counting (weighted spectral counting normalizes mass spectrometric signal to the size of the protein). The data indicated the following: 1) the vast majority of identified proteins were exclusively found in the Gadd45a pulldown sample; 2) most of the proteins that were found in both Gadd45a and control pulldown samples possessed much higher spectral counts in the Gadd45a sample compared with the control sample; and 3) many of the proteins identified in the control sample were actually present at only very low abundance (*i.e.* noise) levels in that sample (supplemental Table 1). As expected, the most abundant protein in the Gadd45a pulldown sample, based on weighted spectral counting, was Gadd45a TAP itself (Fig. 3*A* and supplemental Tables 1–4). Gadd45a TAP was also the most abundant protein in the Gadd45a pulldown sample on the silver-stained gel (Fig. 2).

To identify potential nonspecific interactors in the Gadd45a pulldown sample, we applied a series of three filters to the mass spectrometric data. First, we used weighted spectral counting to estimate differences in protein abundance between the control and Gadd45a pulldown samples. This method indicated that 20 of the proteins identified in the control sample were not significantly (30-fold) enriched in the Gadd45a sample (*i.e.* Gadd45a/control spectral count ratio was <30). Those 20 proteins clearly appeared to be nonspecific interactors, and thus we chose to exclude them from further consideration (Fig. 2; supplemental Table 2).

Our second filter was based upon a previous study that defined some common affinity purification contaminants (21). We identified 19 of those common contaminants in the Gadd45a pulldown sample, as well as 17 proteins that are closely related to the common contaminants (*e.g.* other heat shock proteins and proteosome subunits). We deemed these 36 proteins probable nonspecific interactors and set them aside from further consideration (Fig. 2; supplemental Table 3).

Our final filter was based upon a recent deep proteomics study that estimated the relative abundance of 8309 proteins that are detectable by mass spectrometry in healthy mouse triceps skeletal muscle (22). We reasoned that abundant skeletal muscle proteins were more likely to interact non-specifically with Gadd45a, and thus, we chose to exclude any proteins in the Gadd45a pulldown sample that, on an individual basis, comprised >0.01% of total skeletal muscle protein. In addition, we excluded paralogs of abundant skeletal muscle proteins found in the Gadd45a pulldown sample (*e.g.* other actins and tubulins). This third filter set aside an additional 78 proteins, leaving a stringently defined group of 75 proteins, composed of Gadd45a itself plus 74 low abundance skeletal muscle proteins that appear to specifically interact, directly or indirectly, with Gadd45a *in vivo* (Fig. 2; supplemental Table 4).

Interestingly, of the 74 proteins identified through this workflow, 13 (≈18%) are protein kinases (Fig. 2; supplemental Table 4). These 13 protein kinases, in order of highest to lowest abundance in the Gadd45a pulldown sample, are MEKK4 (also known as MAP3K4 and MTK1), MKK3 (also known as MAP2K3), JAK1, Raf-1, A-Raf, ILK, RSK2, MSK2, MSK1, MKK6 (MAP2K6), RIPK3, MKK7 (MAP2K7), and RIO kinase 3. Fig. 3*A* summarizes the mass spectroscopic data for Gadd45a and associated protein kinases in the Gadd45a pulldown sample. Of note, for three of these kinases, only one unique peptide was identified in the Gadd45a pulldown sample; however, because those kinases are very rare in skeletal muscle (representing <0.001% of total skeletal muscle protein (22)), we suspect they may specifically interact with Gadd45a, albeit at low levels.

##### Gadd45a Interacts with and Activates the Protein Kinase MEKK4 in Skeletal Muscle Fibers

Our overall goal in this study was to discover a direct protein target of Gadd45a that causes skeletal muscle atrophy. The data from our Gadd45a pulldown experiment suggested that Gadd45a may cause skeletal muscle atrophy via interactions with multiple skeletal muscle proteins. Because it was not feasible to investigate all potential Gadd45a targets in the current study, we focused our attention on one, MEKK4. Altogether, in the Gadd45a pulldown sample, we identified 62 unique tryptic peptides covering 41% of the 180-kDa MEKK4 amino acid sequence (Fig. 3*A*). To obtain a more thorough quantitative assessment, we used MS1 filtering (23) (which utilizes peak areas from Skyline MS1 peptide precursor chromatograms (24)) to quantify differences in protein abundance between the control and Gadd45a pulldown samples (supplemental Tables 5 and 6). Fig. 3*B* shows raw data from one representative MEKK4 peptide that was enriched ≈31-fold in the Gadd45a pulldown sample relative to the control pulldown sample (Fig. 3*B*, *left* and *middle panels*), as well as three technical replicates of those data (Fig. 3*B*, *right panel*). Other than Gadd45a itself, MEKK4 was the most abundant protein in the Gadd45a pulldown sample based on weighted spectral counting, and when signals were not normalized for protein size, MEKK4 generated the highest ranking overall search score of any protein in the Gadd45a pulldown sample (Fig. 3*A*).

MEKK4 is a relatively understudied protein in the MAP kinase kinase kinase family. Its role in skeletal muscle had not been previously investigated. However, an elegant series of studies conducted by Saito and co-workers (25–27) over a decade ago demonstrated that MEKK4 can be directly activated by Gadd45a in cultured cell models. To briefly summarize those studies, Saito and co-workers (25–27) found that the kinase domain of MEKK4 resides at the C terminus of the protein; the N-terminal portion of MEKK4 contains an autoinhibitory domain and a Gadd45a-binding site. Under basal conditions, when Gadd45a is not present, MEKK4 exists in an inactive conformation, with its autoinhibitory domain bound to its kinase domain. However, when Gadd45a is present, it directly binds MEKK4 and forces a conformational change that dissociates the autoinhibitory and kinase domains of MEKK4. As a result of this conformational change, the MEKK4 kinase domain becomes active; a specific residue in the MEKK4 kinase activation loop (threonine 1483) is autophosphorylated; and MEKK4 gains the capacity to phosphorylate/activate certain downstream protein kinases, which are currently defined as the MAP kinase kinases MKK3, MKK4, MKK6, and MKK7 (28).

Two pieces of mass spectrometric data from our Gadd45a pulldown sample indicated that Gadd45a not only interacted with MEKK4 but also activated it. First, several MEKK4 peptides were phosphorylated, at serine 116, serine 492, serine 1241, and most importantly, at threonine 1483, the autophosphorylation site in the MEKK4 kinase activation loop (supplemental Files 1–5). Fig. 3*C* shows the tandem mass spectra of a phosphopeptide containing phosphothreonine 1483 in the Gadd45a pulldown sample, and Fig. 3*D* illustrates the location of threonine 1483 within the activation loop of MEKK4. Second, Gadd45a, presumably via MEKK4, interacted with three established MEKK4 substrates, MKK3, MKK6, and MKK7 (Fig. 3*A*). These data strongly suggested that Gadd45a activated MEKK4 in skeletal muscle fibers, consistent with the findings of Saito and co-workers (25–27) in cultured cell models.

**FIGURE 4.**
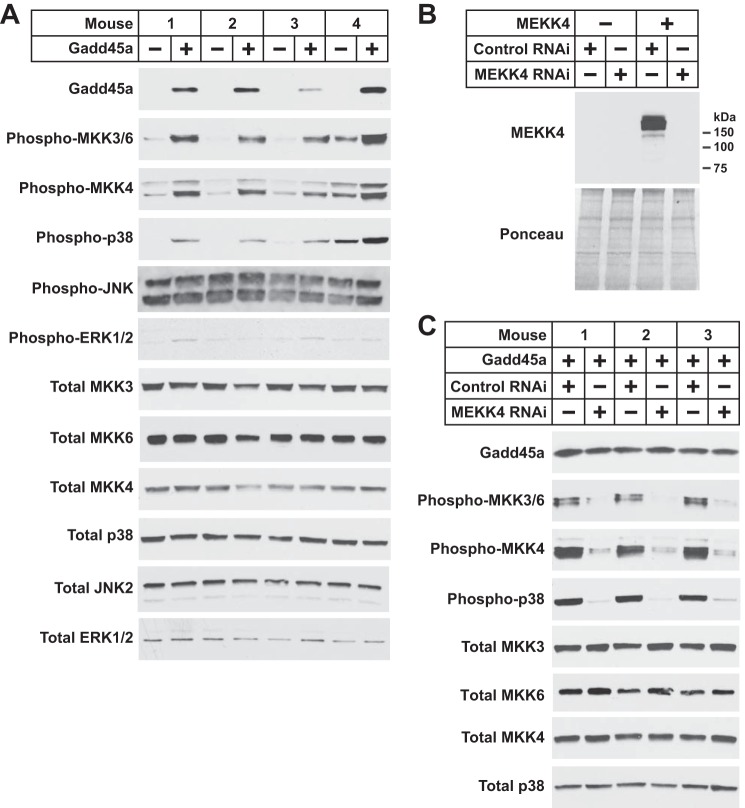
**Gadd45a activates MEKK4 in mouse skeletal muscle.** *A,* one TA per mouse was transfected with 10 μg of empty pcDNA plasmid, and the contralateral TA in each mouse was transfected with 10 μg of Gadd45a-FLAG plasmid, as indicated. Seven days post-transfection, bilateral TA muscles were harvested for immunoblot analysis using the indicated antibodies. *B,* mouse TA muscles were transfected with 5 μg of empty pcDNA plasmid, 5 μg of MEKK4-FLAG plasmid, 20 μg of control RNAi plasmid, and/or 20 μg of MEKK4 RNAi plasmid, as indicated. Seven days post-transfection, TA muscles were harvested for immunoblot analysis using an anti-FLAG antibody. *C,* one TA per mouse was transfected with 10 μg of Gadd45a-FLAG plasmid plus 20 μg of control RNAi plasmid, and the contralateral TA in each mouse was transfected with 10 μg of Gadd45a-FLAG plasmid plus 20 μg of MEKK4 RNAi plasmid, as indicated. Seven days post-transfection, bilateral TA muscles were harvested for immunoblot analysis using the indicated antibodies.

To further investigate whether Gadd45a activates MEKK4 in skeletal muscle, we performed an independent experiment in which we transfected mouse TA muscle fibers *in vivo* with empty plasmid (one TA) or plasmid encoding Gadd45a (contralateral TA). Seven days after transfection, we assessed phosphorylation (*i.e.* activation) of the MEKK4 targets MKK3, MKK6, and MKK4. Of note, the antibody used to detect phosphorylation of MKK3 and MKK6 did not distinguish between MKK3 and MKK6, and we could not assess MEKK4 or MKK7 by immunoblot analysis due to a lack of quality antibodies. Under control conditions, MKK3/6 and MKK4 were minimally phosphorylated (Fig. 4*A*). However, in the presence of Gadd45a, MKK3/6 and MKK4 phosphorylation was markedly increased (Fig. 4*A*). Moreover, Gadd45a-mediated activation of MKK3/6 and MKK4 was accompanied by increased phosphorylation (*i.e.* activation) of p38, a MAP kinase that lies directly downstream of MKK3, MKK4, and MKK6 (Fig. 4*A*). Gadd45a did not affect total levels of MKK3, MKK6, MKK4, or p38, and we did not detect any effects of Gadd45a on the phosphorylation status of JNK and ERK MAP kinases (Fig. 4*A*). Thus, in skeletal muscle, Gadd45a activated certain protein kinases that are known to lie downstream of the Gadd45a-MEKK4 complex, namely MKK3/6, MKK4, and p38.

To determine whether MEKK4 is required for Gadd45a-mediated activation of MKK3/6, MKK4, and p38, we developed an RNA interference (RNAi) construct that specifically reduces MEKK4 protein in mouse skeletal muscle *in vivo* (MEKK4 RNAi; Fig. 4*B*). We then transfected mouse TA muscle fibers *in vivo* with plasmid encoding Gadd45a plus plasmids encoding either a nontargeting control RNAi construct or the MEKK4 RNAi construct. In the presence of nontargeting control RNAi, Gadd45a robustly increased the level of MKK3/6, MKK4, and p38 phosphorylation, as expected (Fig. 4*C*). However, MEKK4 RNAi almost completely abrogated these effects of Gadd45a, without altering the level of Gadd45a or the total amounts of MKK3, MKK6, MKK4, or p38 protein (Fig. 4*C*). These data indicated that MEKK4 is required for Gadd45a-mediated activation of MKK3/6, MKK4 and p38 and provided further evidence that Gadd45a directly activates MEKK4 during skeletal muscle atrophy.

##### MEKK4 Plays an Essential Role in Gadd45a-mediated Skeletal Muscle Atrophy

**FIGURE 5.**
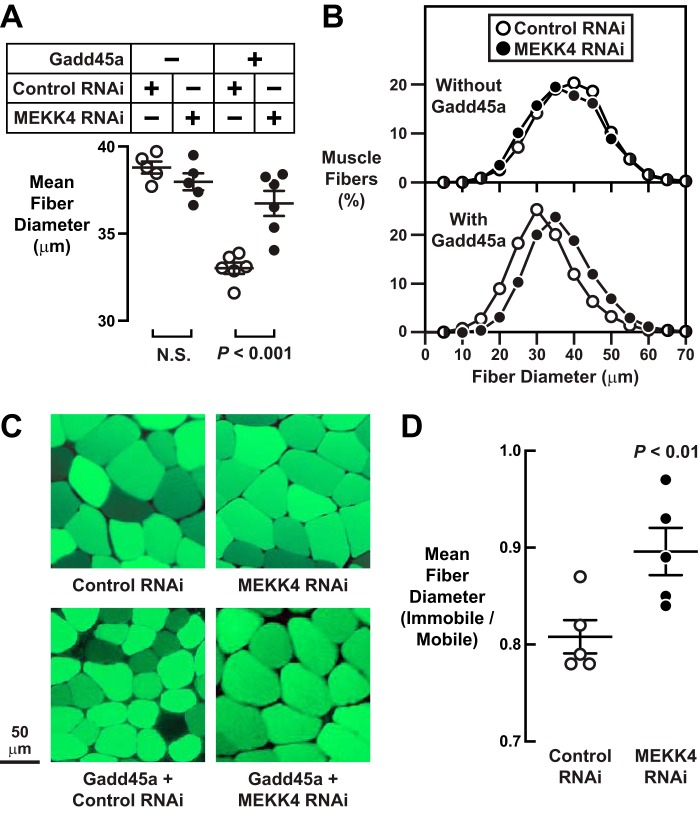
**MEKK4 is required for Gadd45a-mediated skeletal muscle fiber atrophy.** *A–C,* in one cohort of mice, one TA per mouse was transfected with 20 μg of control RNAi plasmid, and the contralateral TA in each mouse was transfected with 20 μg of MEKK4 RNAi plasmid. In a second cohort of mice, one TA per mouse was transfected with 10 μg of Gadd45a-FLAG plasmid plus 20 μg of control RNAi plasmid, and the contralateral TA in each mouse was transfected with 10 μg of Gadd45a-FLAG plasmid plus 20 μg of MEKK4 RNAi plasmid. In all mice, bilateral TA muscles were harvested for histological analysis 7 days post-transfection. *A,* average diameters of skeletal muscle fibers. Each data point represents the mean of >500 muscle fibers from one muscle, and *horizontal bars* denote average of the means ± S.E. *p* values were determined with paired *t* tests. *N.S.,* not significant or *p* > 0.05. *B,* size distribution of all muscle fibers from *A. C,* representative fluorescence microscopy images of muscle cross-sections. *D,* in one cohort of mice, bilateral TA muscles were transfected with 20 μg of control RNAi plasmid. In a second cohort of mice, bilateral TA muscles were transfected with 20 μg of MEKK4 RNAi plasmid. Three days after transfection, one hindlimb in each mouse was immobilized, and then 7 days later (10 days post-transfection), bilateral TA muscles were harvested for histological analysis. In each mouse, the average muscle fiber diameter in the immobilized TA was normalized to the average muscle fiber diameter in the contralateral control (mobile) TA. Each *data point r*epresents the value from one mouse, and *horizontal bars* denote averages ± S.E.

The finding that Gadd45a directly activates MEKK4 during skeletal muscle atrophy suggested the hypothesis that MEKK4 might be required for Gadd45a-mediated skeletal muscle atrophy. To test this hypothesis, we transfected mouse TA muscle fibers *in vivo* with plasmids encoding nontargeting control RNAi (one TA) or MEKK4 RNAi (contralateral TA), both in the absence and presence of Gadd45a plasmid. Seven days after transfection, we harvested bilateral TA muscles for analysis of skeletal muscle fiber size. In the absence of Gadd45a, MEKK4 RNAi did not alter muscle fiber size (Fig. 5, *A–C*), consistent with the notion that it is not possible to reduce MEKK4 activity under basal conditions because it is already inactive, due to the absence of Gadd45a. As expected, the introduction of Gadd45a in the presence of nontargeting control RNAi caused skeletal muscle fiber atrophy (Fig. 5, *A–C*). Importantly, however, MEKK4 RNAi largely reduced Gadd45a-mediated skeletal muscle atrophy (Fig. 5, *A–C*), indicating an essential role for MEKK4.

Immobilization increases skeletal muscle Gadd45a expression and causes skeletal muscle atrophy via a Gadd45a-dependent mechanism (1). To determine whether MEKK4 might be required for immobilization-induced skeletal muscle atrophy, we transfected mouse TA muscle fibers *in vivo* with plasmids encoding either nontargeting control RNAi or MEKK4 RNAi, and then we immobilized the transfected muscles for 7 days. As expected, under control conditions, immobilization caused muscle fiber atrophy, decreasing average muscle fiber diameter by ≈20% (Fig. 5*D*). MEKK4 RNAi significantly reduced the amount of atrophy caused by immobilization, preventing, on average, ≈45% of immobilization-induced muscle fiber atrophy (Fig. 5*D*). These data indicate that MEKK4 plays a significant role in skeletal muscle atrophy caused by at least one common, naturally occurring, Gadd45a-dependent stress condition.

**FIGURE 6.**
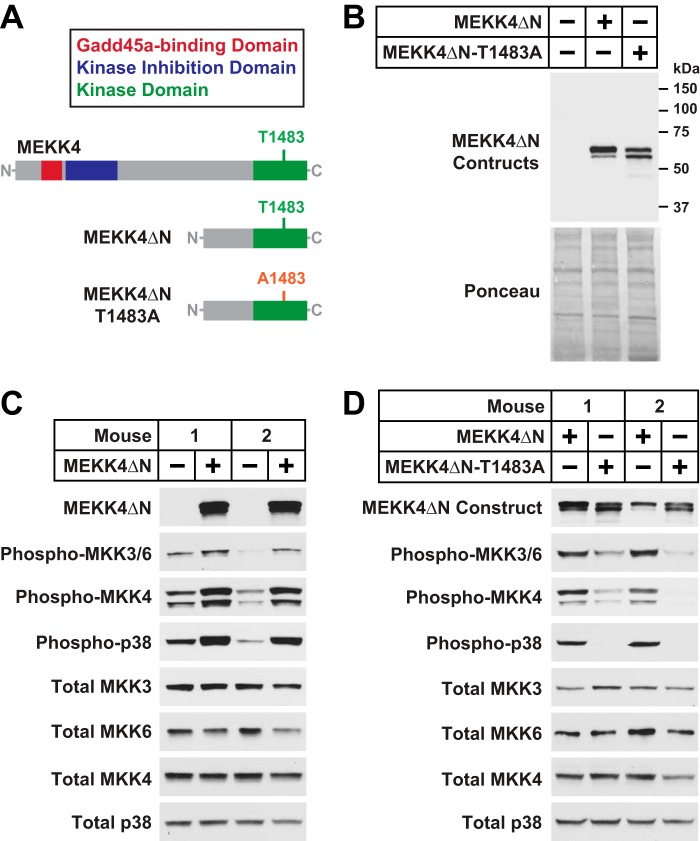
**MEKK4ΔN, a constitutively active Gadd45a-independent MEKK4 construct.** *A,* schematic of the MEKK4ΔN and MEKK4ΔN-T1483A constructs. *B,* TA muscles were transfected with 5 μg of empty pcDNA plasmid, 5 μg of MEKK4ΔN-FLAG plasmid, or 5 μg of MEKK4ΔN-T1483A-FLAG plasmid, as indicated. Three days post-transfection, TA muscles were harvested for immunoblot analysis using monoclonal anti-FLAG IgG. *C,* one TA per mouse was transfected with 5 μg of empty pcDNA plasmid, and the contralateral TA in each mouse was transfected with 5 μg of MEKK4ΔN-FLAG plasmid, as indicated. Three days post-transfection, bilateral TA muscles were harvested for immunoblot analysis using the indicated antibodies. *D,* one TA per mouse was transfected with 5 μg of MEKK4ΔN-FLAG plasmid, and the contralateral TA in each mouse was transfected with 5 μg of MEKK4ΔN-T1483A-FLAG plasmid, as indicated. Three days post-transfection, bilateral TA muscles were harvested for immunoblot analysis using the indicated antibodies.

To investigate whether MEKK4 activity might be sufficient to induce skeletal muscle atrophy, we generated a constitutively active Gadd45a-independent MEKK4 construct that retains the C-terminal kinase domain but lacks the N-terminal autoinhibitory and Gadd45a binding domains (26, 29). This construct, termed MEKK4ΔN, is illustrated in Fig. 6, *A* and *B*. As a control for potential kinase-independent effects of MEKK4ΔN, we also generated an inactive MEKK4ΔN construct in which the autophosphorylation site in the kinase activation loop, threonine 1483, is mutated to alanine (MEKK4ΔN-T1483A; Fig. 6, *A* and *B*). When transfected into mouse skeletal muscle *in vivo*, the MEKK4ΔN plasmid strongly induced MKK3/6, MKK4, and p38 phosphorylation in a Gadd45a-independent manner (Fig. 6*C*), and these effects were abolished by the T1483A mutation (Fig. 6*D*). Thus, MEKK4ΔN is constitutively active in mouse skeletal muscle fibers, whereas MEKK4ΔN-T1483A is inactive.

**FIGURE 7.**
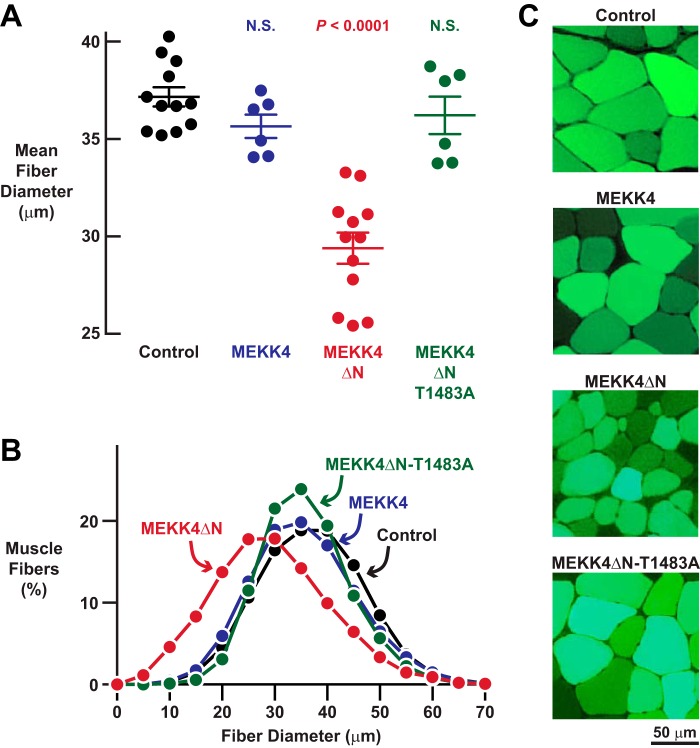
**MEKK4ΔN induces skeletal muscle fiber atrophy in a Gadd45a-independent manner.** *A–C,* mouse TA muscles were transfected with 5 μg of empty pcDNA plasmid, 5 μg of MEKK4-FLAG plasmid, 5 μg of MEKK4ΔN-FLAG plasmid, and/or 5 μg of MEKK4ΔN-T1483A-FLAG plasmid, as indicated. All muscles were co-transfected with 2.5 μg of eGFP plasmid. TA muscles were harvested for histological analysis 7 days post-transfection. *A,* average diameters of skeletal muscle fibers. Each data point represents the mean of >400 muscle fibers from one muscle, and *horizontal bars* denote average of the means ± S.E. *p* values were determined with a one-way ANOVA and Dunnett's multiple comparison test. *N.S.,* not significant or *p* > 0.05. *B,* size distribution of all muscle fibers from *A. C,* representative fluorescence microscopy images of muscle cross-sections.

To determine the effects of MEKK4 activity on skeletal muscle fiber size, we transfected mouse TA muscle fibers with plasmids encoding either MEKK4ΔN or MEKK4ΔN-T1483A. As additional controls, we transfected muscle fibers with empty plasmid or plasmid encoding full-length MEKK4 (an inactive kinase in the absence of Gadd45a). We found that MEKK4ΔN induced a dramatic loss of muscle fiber size (Fig. 7, *A–C*). In contrast, full-length MEKK4 and MEKK4ΔN-T1483A did not significantly alter muscle fiber size (Fig. 7, *A–C*). These data indicate that MEKK4 activity is sufficient to induce skeletal muscle fiber atrophy and provide further evidence that Gadd45a causes skeletal muscle atrophy at least in part by activating MEKK4.

## Discussion

In this study, we sought to better understand how Gadd45a causes skeletal muscle atrophy. To that end, we used a biochemical approach to identify skeletal muscle proteins that associate with Gadd45a as it induces skeletal muscle atrophy *in vivo*. Through this work, we discovered multiple skeletal muscle proteins that interact with Gadd45a in skeletal muscle fibers. Furthermore, we found that at least one of these proteins, MEKK4, plays an important role in Gadd45a-mediated skeletal muscle atrophy.

**FIGURE 8.**
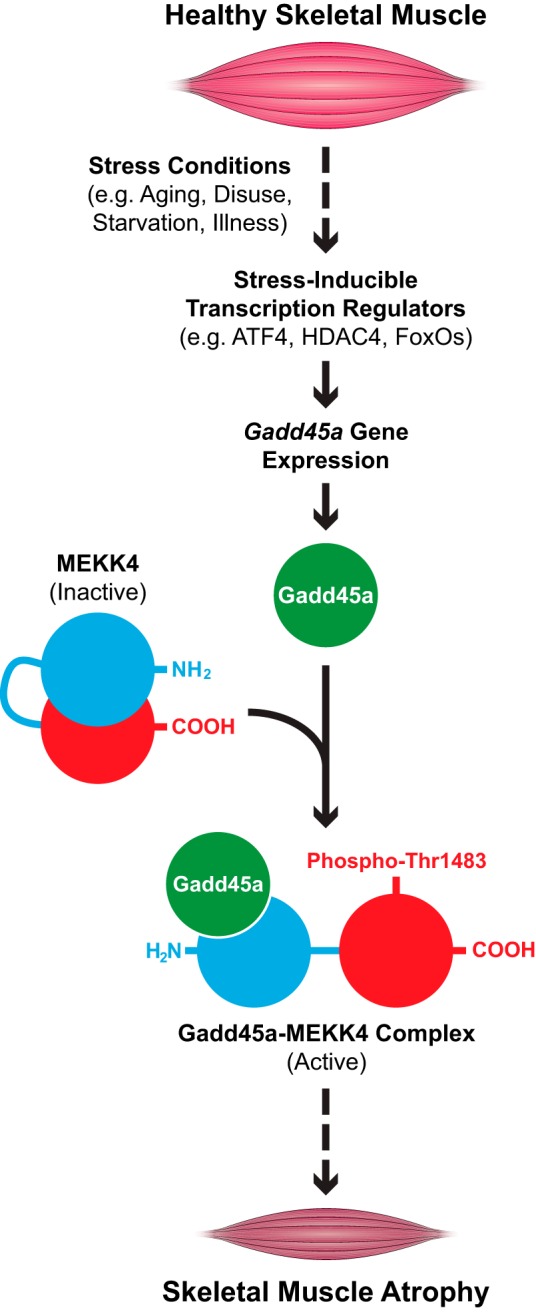
**Proposed model illustrating the role of MEKK4 in Gadd45a-mediated skeletal muscle atrophy.**

These data, added to previous findings, suggest a model that is illustrated in Fig. 8. In healthy skeletal muscle fibers, the *Gadd45a* gene is inactive, and thus, Gadd45a protein levels are low. Under these circumstances, the MEKK4 protein exists in an inactive conformation, with its C-terminal kinase domain occluded by its N-terminal autoinhibitory domain. Our data suggest that a low level of MEKK4 activity in skeletal muscle fibers is necessary to maintain normal fiber size.

The situation changes for the worse when animals experience starvation, muscle disuse, severe systemic illness or aging (Fig. 8). These stress conditions activate certain transcription regulators in skeletal muscle fibers (ATF4, HDAC4, and/or FoxOs) that generate Gadd45a protein by inducing the *Gadd45a* gene. Gadd45a then binds the N-terminal region of MEKK4, relieving autoinhibition, stimulating autophosphorylation of threonine 1483, and generating an active Gadd45a-MEKK4 kinase complex that is sufficient to induce skeletal muscle fiber atrophy.

The mechanism by which the Gadd45a-MEKK4 complex promotes skeletal muscle atrophy is not yet known. It seems most obvious to us that the complex causes atrophy by phosphorylating one or more downstream proteins, but we do not currently know what those proteins are. Our data indicate that, in skeletal muscle fibers, the Gadd45a-MEKK4 complex binds and/or activates all four of its previously established downstream MAP kinase kinases (MKK3, MKK4, MKK6, and MKK7), leading to activation of one or more p38 MAP kinases (*i.e.* p38α, β, γ, and/or δ). Based on these observations, we speculate that MKK3, MKK4, MKK6, MKK7, and/or one or more p38 kinases may be important downstream mediators of the Gadd45a-MEKK4 complex in skeletal muscle fibers; yet it also remains possible that the Gadd45a-MEKK4 complex may induce muscle atrophy by affecting proteins that are not yet known to lie downstream of the complex. It is also important to note that Gadd45a may promote muscle atrophy by MEKK4-independent mechanisms, which may involve some of the other Gadd45a-interacting proteins identified here. These are important areas for future investigation.

At this point, it is uncertain to us whether the Gadd45a-MEKK4 complex might be a reasonable drug target. Although pharmacological inhibition of this complex would be predicted to reduce skeletal muscle atrophy, it might also have untoward effects in other tissues. For example, studies of global MEKK4 knock-out mice indicate that a targeted and complete whole body loss of MEKK4 activity reduces viability and impairs T cell signaling, cerebral cortical development, and neural tube formation (30–32). Nonetheless, the current results could recommend a search for potential drug targets that lie downstream of the Gadd45a-MEKK4 complex in skeletal muscle fibers, as well as pharmacological agents that act upstream of the complex to blunt its activity in skeletal muscle.

In our Gadd45a pulldown experiment, we stringently filtered the data in an attempt to reduce false-positive results. We do not know whether those filters may have eliminated certain proteins that truly interact with Gadd45a in skeletal muscle fibers and/or failed to eliminate some false-positive interactors. Thus, we believe that future studies in this area should consider both the unfiltered and filtered data, which are fully provided in the supplemental Tables. In addition, it is not possible for us to know right now whether technical aspects of our protocol may have prevented us from capturing some proteins that truly interact with Gadd45a in skeletal muscle fibers. Notwithstanding these methodological limitations, this study provides proof-of-concept for a new approach to discover protein-protein interactions in skeletal muscle *in vivo*. This general experimental approach could be applied to further investigations of Gadd45a or other skeletal muscle proteins of interest.

In summary, this current study identifies a direct biochemical mechanism by which Gadd45a promotes skeletal muscle fiber atrophy. These results provide new insight into the way that skeletal muscle atrophy occurs at the molecular level.

## Experimental Procedures

### 

#### 

##### Chemicals

3× FLAG peptide was from Sigma (catalog no. F4799). HPLC solvents, including acetonitrile and water, were obtained from Burdick & Jackson (Muskegon, MI). Reagents for protein chemistry, including iodoacetamide, dithiothreitol (DTT), ammonium bicarbonate, formic acid, and urea, were purchased from Sigma. Proteomics grade trypsin was from Promega (Madison WI). HLB Oasis SPE cartridges were purchased from Waters (Milford, MA).

##### Buffers

Buffer A was 50 mm HEPES, pH 7.0, 150 mm NaCl, 5 mm MgCl_2_, 2 mm EGTA, 0.5% (v/v) Triton X-100, and 0.5 mm DTT. Buffer B was 50 mm HEPES, pH 7.0, 150 mm NaCl, 5 mm MgCl_2_, 2 mm EGTA, 0.1% (v/v) Triton X-100, and 0.5 mm DTT. Buffer C was 50 mm HEPES, pH 7.0, 150 mm NaCl, 5 mm MgCl_2_, 2 mm EGTA, 0.1% Triton X-100, 1% SDS, and 0.5 mm DTT. All buffers were made with ultra-pure water (Gibco). Immediately before use, all buffers were supplemented with cOmplete mini EDTA-free protease inhibitor (Roche Applied Science) and PhosStop phosphatase inhibitor (Roche Applied Science) according to the manufacturer's instructions.

##### Antibodies

Anti-FLAG M2 magnetic beads were from Sigma (catalog no. M8823). S-Protein agarose was from EMD Millipore (catalog no. 69704). Mouse monoclonal anti-FLAG IgG was from Sigma (catalog no. F1804). The following antibodies were from Cell Signaling: rabbit monoclonal anti-MKK3 IgG (catalog no. 8535); rabbit monoclonal anti-MKK6 IgG (catalog no. 8550); rabbit monoclonal anti-phospho-MKK3(Ser-189)/MKK6(Ser-207) IgG (catalog no. 12280); rabbit polyclonal anti-MKK4 antibody (catalog no. 9152); rabbit monoclonal anti-phospho-MKK4(Ser-257) antibody (catalog no. 4514); rabbit polyclonal anti-p38 MAPK antibody (catalog no. 9212); rabbit monoclonal anti-phospho-p38(Thr-180/Tyr-182) IgG (catalog no. 4511); rabbit monoclonal anti-JNK2 antibody (catalog no. 9258); rabbit monoclonal anti-phospho-JNK(Thr-183/Tyr-185) IgG (catalog no. 4671); rabbit monoclonal anti-ERK1/2 IgG (catalog no. 4695); rabbit monoclonal anti-phospho-ERK1/2(Thr-202/Tyr-204) IgG (catalog no. 4370); HRP-conjugated anti-mouse IgG (catalog no. 7076); and HRP-conjugated anti-rabbit IgG (catalog no. 7074).

##### Plasmids

The Gadd45a-FLAG plasmid has been described previously (1) and encodes mouse Gadd45a with three copies of the FLAG epitope tag at the N terminus, under control of the cytomegalovirus (CMV) promoter. The eGFP plasmid encodes eGFP under control of the CMV promoter. The empty TAP plasmid was described previously (as pSS-FS plasmid (33)). The Gadd45a TAP plasmid was generated by PCR amplifying the Gadd45a coding region from the Gadd45a-FLAG plasmid and then cloning that fragment into the empty TAP plasmid. The control RNAi plasmid was described previously (1) and encodes emerald green fluorescent protein (EmGFP) and a nontargeting pre-miRNA under bicistronic control of the CMV promoter in the pcDNA6.2GW/EmGFP-miR plasmid (Invitrogen). The MEKK4 RNAi plasmid encodes EmGFP and an artificial pre-miRNA targeting mouse MEKK4 under bicistronic control of the CMV promoter; it was generated by ligating the Mmi538666 oligonucleotide duplex (Invitrogen) into the pcDNA6.2GW/EmGFP-miR plasmid. The MEKK4-FLAG plasmid encodes mouse MEKK4 with three copies of the FLAG epitope tag at the C terminus, under control of the CMV promoter; it was generated by subcloning a full-length mouse MEKK4 cDNA (ATCC clone Mm5705378) into the p3× FLAG-CMV-14 plasmid (Sigma). The MEKK4ΔN-FLAG plasmid encodes a start methionine followed by amino acids 1051–1597 of mouse MEKK4 followed by three copies of the FLAG epitope tag at the C terminus, under control of the CMV promoter; it was generated by PCR amplification from the full-length MEKK4 and then cloning into the p3× FLAG-CMV14 plasmid. The MEKK4ΔN-T1483A-FLAG plasmid encodes MEKK4ΔN-FLAG with a threonine-to-alanine mutation at the residue equivalent to threonine 1483 in full-length mouse MEKK4; it was generated by site-directed mutagenesis of the MEKK4ΔN-FLAG plasmid. All plasmids were verified by sequencing the entire coding region of the plasmid.

##### Mouse Protocols

The mice used in these studies were males from the C57BL/6 strain, obtained from the National Cancer Institute (National Institutes of Health) at ages 6–8 weeks and used for experiments within 2 weeks of their arrival. Animals were housed in colony cages at 21 °C with 12-h light/12-h dark cycles and had *ad libitum* access to standard chow (Harlan Teklad formula 7013) and water throughout the study. Transfection of mouse skeletal muscle with plasmid DNA was performed as described previously (12); briefly, mice were anesthetized with ketamine/xylazine, hindlimbs were shaved, and the TA muscles were injected with 30 μl of 0.4 units/μl bovine placental hyaluronidase (Sigma) resuspended in sterile 0.9% saline. Two hours later, mice were re-anesthetized. The TA muscles were then injected with 30 μl of plasmid DNA in sterile saline, coated with ultrasound jelly, and subjected to 10 20-ms pulses of 175 V/cm using an ECM-830 electroporator (BTX Harvard Apparatus). Following transfection, mice were returned to their cages to resume normal activities for 3–10 days before muscle harvest, as noted in the figure legends. Unilateral TA muscle immobilization was performed as described previously (1). All animal procedures were approved by the Institutional Animal Care and Use Committee of the University of Iowa.

##### Immunoblot Analyses

Protein extracts from mouse skeletal muscle were prepared as described previously (1). An aliquot of each protein extract was then mixed with 0.25 volume of sample buffer (1) and heated for 5 min at 95 °C. A separate aliquot of each extract was used to determine protein concentration by the BCA kit, after which an equivalent amount of protein from each sample was subjected to SDS-PAGE. For immunoblot analysis, proteins were transferred from SDS-polyacrylamide gels to 0.45 nitrocellulose membranes (Bio-Rad catalog no. 162-0115). Immunoblots were performed at 4 °C for 16 h using primary antibodies diluted to 1:1000 (anti-MKK3, anti-MKK6, anti-phospho-MKK3/6, anti-MKK4, anti-phospho-MKK4, anti-p38, anti-phospho-p38, anti-JNK, and anti-phospho-JNK), 1:2000 (anti-ERK1/2 and anti-phospho-ERK1/2), or 1:4000 (anti-FLAG). Bound antibodies were visualized by chemiluminescence (SuperSignal West Pico; Thermo Scientific) using HRP-conjugated anti-mouse or anti-rabbit IgG. Membranes were stained with Ponceau S to confirm equal loading.

##### Histological Analysis of Skeletal Muscle Fibers

Harvested muscles were immediately fixed in 4% (w/v) paraformaldehyde for 16 h at 4 °C and then incubated in 30% sucrose (w/v) for 24 h. The muscles were then embedded in Tissue Freezing Medium (Triangle Biomedical Sciences), and a Microm HM 505E cryostat was used to prepare 10-μm sections from the muscle midbelly. Sections were washed with PBS three times and then mounted with Vectashield (Vector Laboratories). All sections were examined and photographed using an Nikon Eclipse Ti automated inverted microscope equipped with NIS-Elements BR digital imaging software. Image analysis was performed using ImageJ software. Skeletal muscle fiber size was analyzed by measuring the lesser diameter (minimal *Feret* diameter) of muscle fibers, as recommended elsewhere (34).

##### Tandem Affinity Purification

Freshly excised mouse TA muscles were immediately frozen in liquid N_2_ and stored at −80 °C. To prepare protein lysates, groups of four TA muscles transfected with either empty TAP plasmid or Gadd45a TAP plasmid were placed in 17 × 100-mm Falcon tubes containing 2 ml of ice-cold buffer A and then homogenized with an OMNI TH-01 tissue tearer. Homogenates were then moved to 2-ml microcentrifuge tubes, rotated for 2 h at 4 °C, and then centrifuged at 12,000 × *g* for 30 min. The soluble fractions from each group (control and Gadd45a) were then pooled in one 15-ml conical tube per group. We then added 0.6 ml of pre-washed anti-FLAG magnetic beads to each sample, rotated the samples for 2 h at 4 °C, captured the magnetic beads with a magnetic separator, removed and discarded the supernatant, and briefly washed the beads four times on ice with 6 ml of ice-cold buffer B. To elute bound proteins, we incubated and rotated the beads in 3 ml of buffer B containing 450 μg of 3× FLAG peptide for 30 min at 4 °C, captured the beads with a magnetic separator, moved the supernatant to a fresh 15-ml tube on ice, briefly incubated the beads two more times with 3 ml of ice-cold buffer B, and combined these two additional supernatants with the first supernatant containing 3× FLAG peptide. We then added 0.4 ml of pre-washed S-protein agarose to the two samples (control and Gadd45a), incubated the samples on a belly shaker for 2 h at 4 °C, centrifuged the samples at 200 × *g* for 1 min, briefly washed the pellets three times on ice with 1.2 ml of ice-cold buffer B, resuspended the pellets in 1.2 ml of ice-cold buffer B, transferred the samples to 1.5-ml of LoBind microcentrifuge tubes (Eppendorf), and centrifuged at 200 × *g* for 1 min. We then removed and discarded the supernatants, resuspended the pellets in 0.4 ml of ice-cold buffer C, incubated the samples for 5 min at 65 °C, centrifuged the samples at 200 × *g* for 1 min, and then transferred the supernatants to fresh 1.5 ml of LoBind microcentrifuge tubes on ice. We then repeated this process two more times (resuspending the beads in 0.4 ml of ice-cold buffer C, incubating them for 5 min at 65 °C, and centrifuging them at 200 × *g* for 1 min), each time transferring the resulting supernatant to the first supernatant in the 1.5-ml microcentrifuge tube on ice. The two pulldown samples (each containing the combined final supernatants from either control or Gadd45a muscles) were then centrifuged at 13,000 × *g* for 1 min at 4 °C to remove any residual beads, and the resulting supernatants were then concentrated to 75 μl with Ultra Ultracel-10K columns (Amicon). We then added 25 μl of NuPAGE 4× LDS sample buffer to each sample and stored the samples at −80 °C. An aliquot of each pulldown sample (10 μl) was subsequently incubated for 10 min at 65 °C, subjected to SDS-PAGE on 4–12% NuPAGE gels (Invitrogen), and then visualized with SilverQuest silver staining kit (Invitrogen). The remainder of each pulldown sample (90 μl) was used for mass spectrometric studies, as described below.

##### Preparation of Pulldown Samples for Mass Spectrometry

Two gel process replicates of control and Gadd45a TAP pulldown samples were processed for mass spectrometric analysis. To remove detergents and other non-compatible reagents, protein samples were subjected to a “stack” gel clean-up procedure as follows: after TAP pulldown and elution, samples were added to 10 μl of NuPAGE LDS sample buffer and incubated at 70 °C for 10 min. Samples were then loaded into a NuPAGE 4–12% BisTris gel, run only until the dye front had progressed about 1 cm into the gel, and visualized with GelCode Blue Stain Reagent (Life Technologies, Inc.). Subsequently, three contiguous gel slices were excised from the 1-cm-long, protein-containing gel range, destained, reduced with a final concentration of 10 mm DTT at 56 °C, and alkylated with 55 mm iodoacetamide at room temperature in the dark. In-gel trypsin digestion was performed using a 1:20 enzyme to protein ratio overnight at 37 °C. Resulting peptides were extracted and desalted.

##### Mass Spectrometry

Samples were analyzed by reverse-phase HPLC-ESI-MS/MS using an Eksigent Ultra Plus nano-LC two-dimensional HPLC system (Dublin, CA) connected to a quadrupole time-of-flight TripleTOF 5600 (QqTOF) mass spectrometer (SCIEX). Typically, mass resolution for MS1 scans and corresponding precursor ions was ∼35,000 (TripleTOF 5600), and resolution for MS/MS scans and resulting fragment ions (MRM-HR transitions) was ∼15,000 (“high sensitivity” product ion scan mode) (23). Briefly, after injection, peptide mixtures were transferred onto the analytical C18-nanocapillary HPLC column (C18 Acclaim PepMap100, 75 μm inner diameter × 15 cm, 3-μm particle size, 100-Å pore size, Dionex, Sunnyvale, CA) and eluted at a flow rate of 300 nl/min using stepwise gradients from 5 to 80% solvent B with total run times, including mobile phase equilibration, of 90 min. Solvent mobile phase A was 2% acetonitrile, 98% of 0.1% formic acid (v/v) in water, and mobile phase B was 98% acetonitrile, 2% of 0.1% formic acid (v/v) in water. Data acquisition was performed in data-dependent acquisition (DDA) mode on the TripleTOF 5600 to obtain MS/MS spectra for the 30 most abundant precursor ions (50 ms per MS/MS) following each survey MS1 scan (250 msec) yielding a total cycle time of 1.8 s as described previously (23, 35). Each of the process (pulldown) replicates was acquired in three technical replicates (MS injection replicates).

##### Bioinformatic Database Searches

Mass spectrometric data were searched using the database search engine Protein Pilot (36) (SCIEX Beta 4.5, revision 1656) with the Paragon algorithm (4.5.0.0, 1654). The search parameters were set as follows: trypsin digestion, cysteine alkylation set to iodoacetamide, and *Mus musculus* as species. Additional searches utilized phosphorylation emphasis. Trypsin specificity was assumed as C-terminal cleavage at lysine and arginine. Processing parameters were set to “biological modification,” and a thorough identification search effort was used. For database searches, a cutoff peptide confidence value of 99 was chosen, and a minimum of two identified peptides per protein was required. Five phosphopeptide MS/MS spectra and 10 selected “one peptide wonder” MS/MS from other kinases and phosphatases were inspected for quality, and the corresponding spectral library was uploaded to Panorama as described below. Briefly, the MS/MS spectra were inspected “manually” based on an adaptation of previously published criteria (37). Proteins with only one observed peptide were validated and added to the list only if the following criteria were satisfied. 1) The peptide represented a unique peptide in the human proteome as confirmed by BLAST searches. 2) Manual inspection of the spectrum verified the following criteria: (*a*) good signal-to-noise (S/N >3, for the majority of fragment ions); (*b*) a minimum of three consecutive y- or b-ions; and (*c*) any y-ion with an N-terminal proline was intense. The Protein Pilot false discovery rate (FDR) analysis tool, the Proteomics System Performance Evaluation Pipeline (PSPEP algorithm) (36), provided a global FDR of 1% and a local FDR at 1% in all cases. All database search results and details for peptide identifications are provided in supplemental Tables 7–9. Tandem mass spectra (MS/MS) for identified phosphorylated peptides from protein MEKK4 are shown in supplemental Files 1–5.

##### Quantitative Assessments and Data Analysis-Spectral Counting

To estimate protein levels in the control and Gadd45a pulldown samples, mass spectrometric acquisitions were initially subjected to spectral counting (38) and weighted spectral counting (39) with a weighted spectral counting method chosen accounting for theoretically possible tryptic peptides to be generated from a given protein. Data from the control and Gadd45a pulldown samples were obtained from two SDS-PAGE process replicates, and for each process replicate three technical MS injection replicates were acquired. For the spectral counting assessments, data were combined for all control acquisitions, and separately data were combined for all Gadd45a TAP acquisitions.

##### Quantitative Skyline MS1 Filtering

MS1 (peptide precursor) chromatogram-based quantification was performed in Skyline 2.5 (24), an open source software project, as recently described in detail for MS1 filtering (23). Briefly, first, comprehensive spectral libraries were generated in Skyline from database searches of the raw data files prior to MS1 filtering. Second, all raw files acquired in DDA mode from two process and three technical replicates per sample type (control and Gadd45a TAP) were imported into Skyline 2.5, and MS1 precursor ions were extracted for all peptides present in the MS/MS spectral libraries. Quantitative MS1 analysis is based on extracted ion chromatograms and resulting precursor ion peak areas for M, M + 1, and M + 2. Final quantitative comparisons are typically based on only the highest ranked precursor ion, and results were exported from Skyline for the control and Gadd45a samples (see supplemental Tables 5 and 6). In all cases, significance was assessed using two-tailed Student's *t* test (*p* < 0.05).

##### Raw Data Accession

The mass spectrometric raw data, and spectral libraries associated with this study may be downloaded from MassIVE at ftp://massive.ucsd.edu/MSV000079730 (MassIVE ID: MSV000079730) or from ProteomeXchange with the accession number PXD004169). A Skyline spectral library of identified phosphorylated peptides from MEKK4 and selected “One Peptide Wonders” from other kinases were uploaded to Panorama Public (https://panoramaweb.org/labkey/Gadd45a.url), an interactive web-based repository for sets processed with Skyline (40).

##### Modeling of Kinase Domain of MEKK4

The Protein Model Portal was used to model the kinase domain (residues 1337–1591) of mouse MEKK4 (SwissProt accession number O08648). The model is based on Protein Data Bank template 3comA (crystal structure of Mst1 kinase, sequence identity 33%). Quality criteria indicate whether the model is considered reliable (*green*) or unreliable (*red*) as shown in Fig. 3*D.*

## Author Contributions

S. A. B. coordinated the experiments and performed them with invaluable contributions from S. S., M. C. D., J. M. D., S. M. E., and A. D. D., B. S., and B. W. G. performed the mass spectrometry analyses. C. M. A. conceived the study, designed the experiments, and wrote the paper. All authors reviewed the results, contributed to data analysis and manuscript preparation, and approved the final version of the manuscript.

## Supplementary Material

Supplemental Data

## Footnotes

2 The abbreviations used are:

TAP: tandem affinity purification
TA: tibialis anterior
eGFP: enhanced GFP
EmGFP: emerald GFP
BisTris: 2-[bis(2-hydroxyethyl)amino]-2-(hydroxymethyl)propane-1,3-diol
MAP: mitogen-activated protein
FDR: false discovery rate.
