# Supplementary material for: Gadd45a Protein Promotes Skeletal Muscle Atrophy by Forming a Complex with the Protein Kinase MEKK4
Source: J Biol Chem. 2016 Jun 29;291(34):17496–509. doi: 10.1074/jbc.M116.740308 (PMC5016147; doi:10.1074/jbc.M116.740308)
Supplement: Supplemental Data [file supp_291_34_17496__index.html]

Gadd45a Promotes Skeletal Muscle Atrophy by Forming a Complex with the Protein Kinase MEKK4 — Gadd45a Protein Promotes Skeletal Muscle Atrophy by Forming a Complex with the Protein Kinase MEKK4 — Gadd45a-MEKK4 Complex Promotes Skeletal Muscle Atrophy — Supplemental Data 

# Gadd45a Protein Promotes Skeletal Muscle Atrophy by Forming a Complex with the Protein Kinase MEKK4

## Supplemental Data

- Supplemental Tables 1-9 (.xlsx, 421 KB) - Proteomics Data
- Supplemental Files 1-5 (.pptx, 142 KB) - Proteomics Data
