# Supplementary material for: Gadd45a Protein Promotes Skeletal Muscle Atrophy by Forming a Complex with the Protein Kinase MEKK4
Source: J Biol Chem. 2016 Jun 29;291(34):17496–509. doi: 10.1074/jbc.M116.740308 (PMC5016147; doi:10.1074/jbc.M116.740308)
Supplement: Supplemental Data [file 10.1074_M116.740308_jbc.M116.740308-2.pptx]

## Slide 1
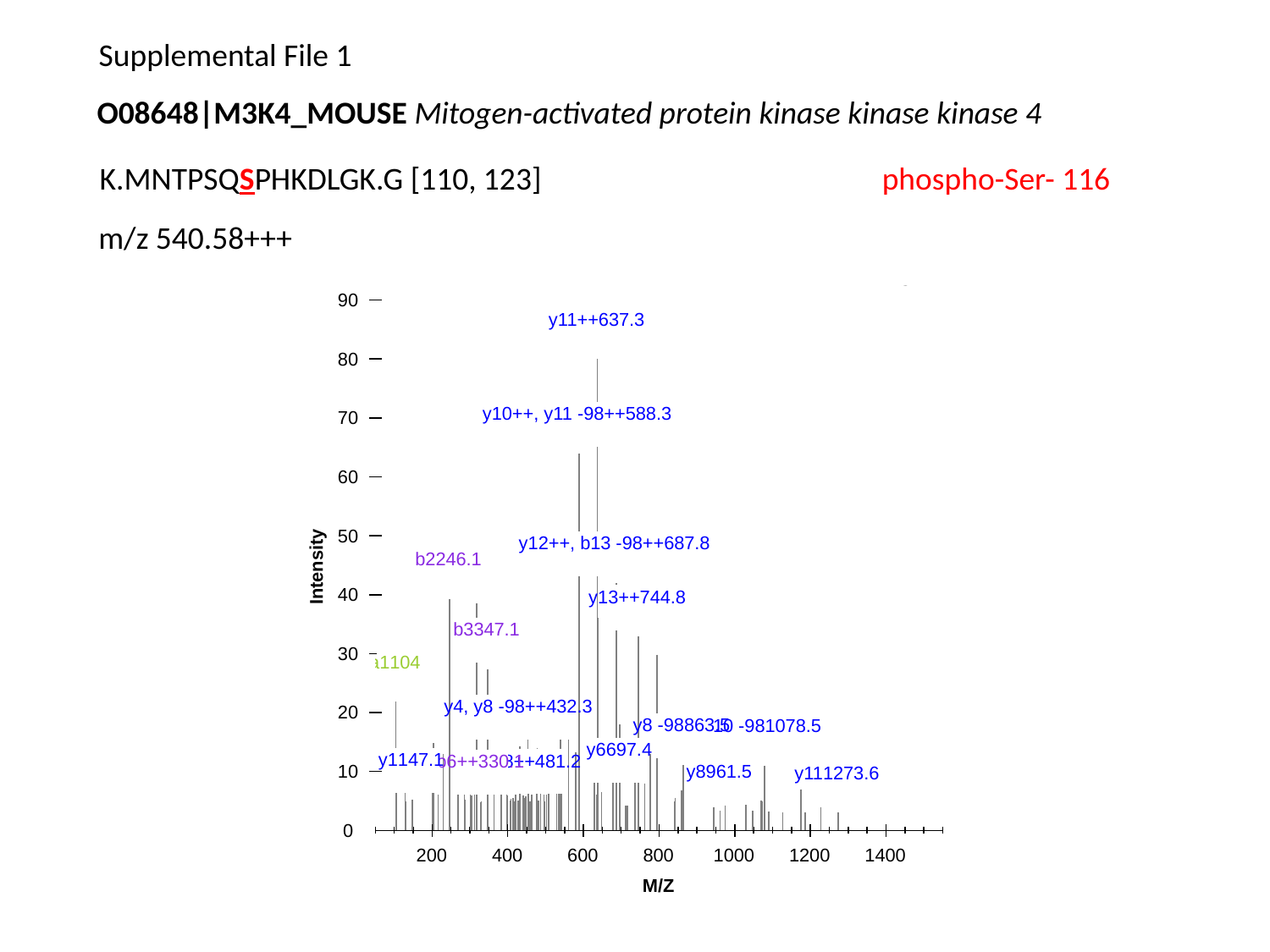

Supplemental File 1
O08648|M3K4_MOUSE Mitogen-activated protein kinase kinase kinase 4
K.MNTPSQSPHKDLGK.G [110, 123]
phospho-Ser- 116
m/z 540.58+++

## Slide 2
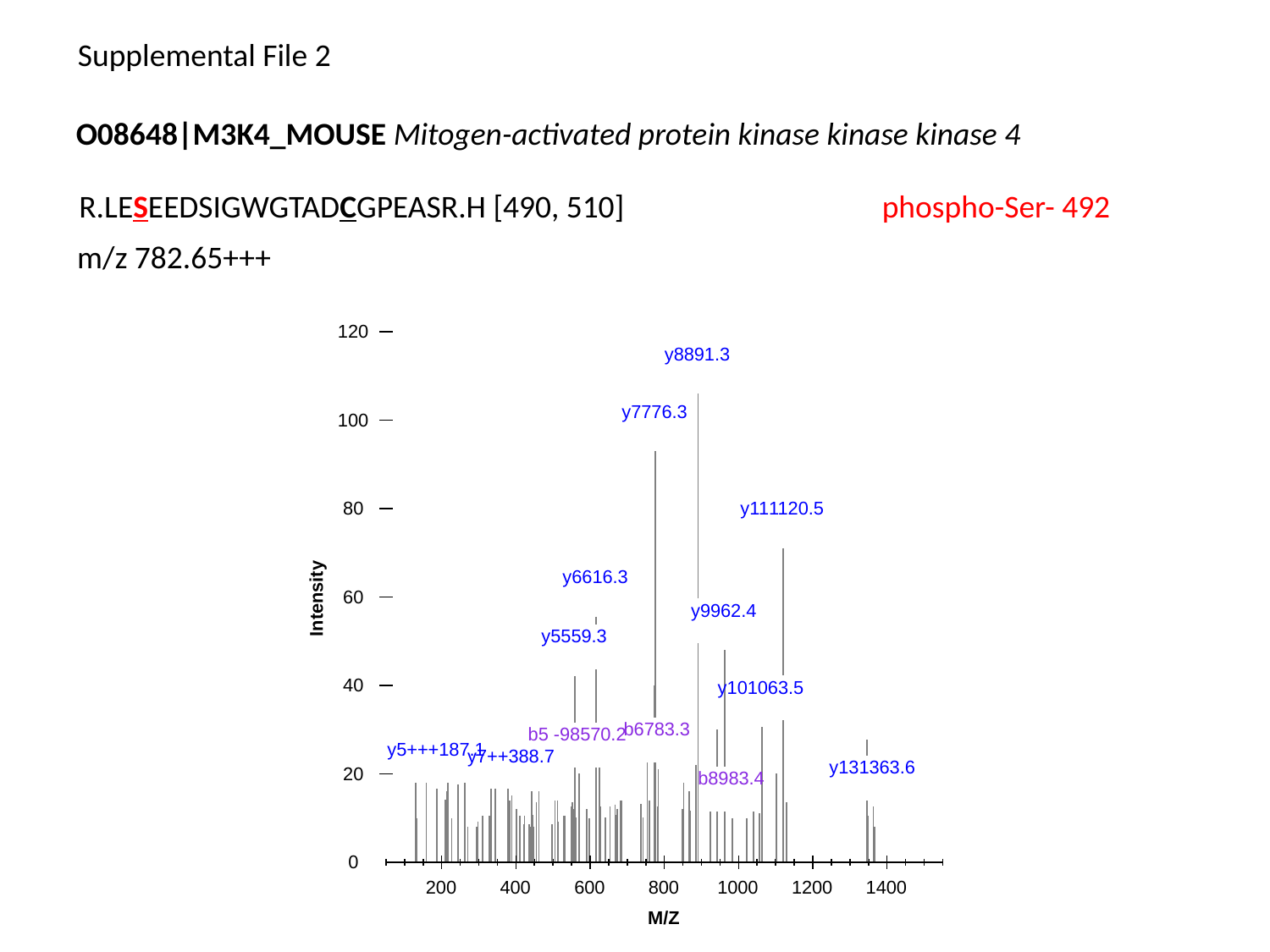

Supplemental File 2
O08648|M3K4_MOUSE Mitogen-activated protein kinase kinase kinase 4
R.LESEEDSIGWGTADCGPEASR.H [490, 510]
phospho-Ser- 492
m/z 782.65+++

## Slide 3
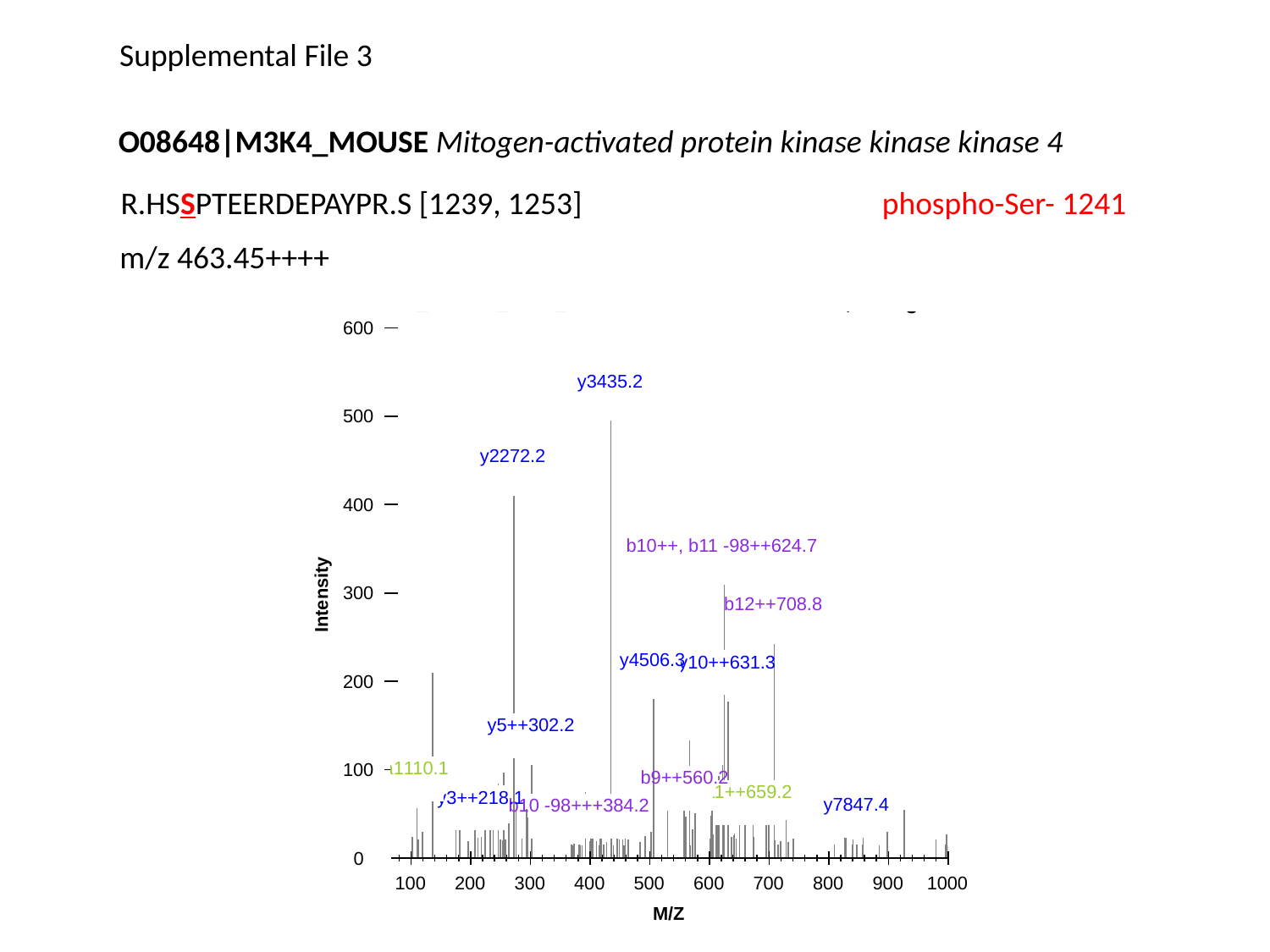

Supplemental File 3
O08648|M3K4_MOUSE Mitogen-activated protein kinase kinase kinase 4
R.HSSPTEERDEPAYPR.S [1239, 1253]
phospho-Ser- 1241
m/z 463.45++++

## Slide 4
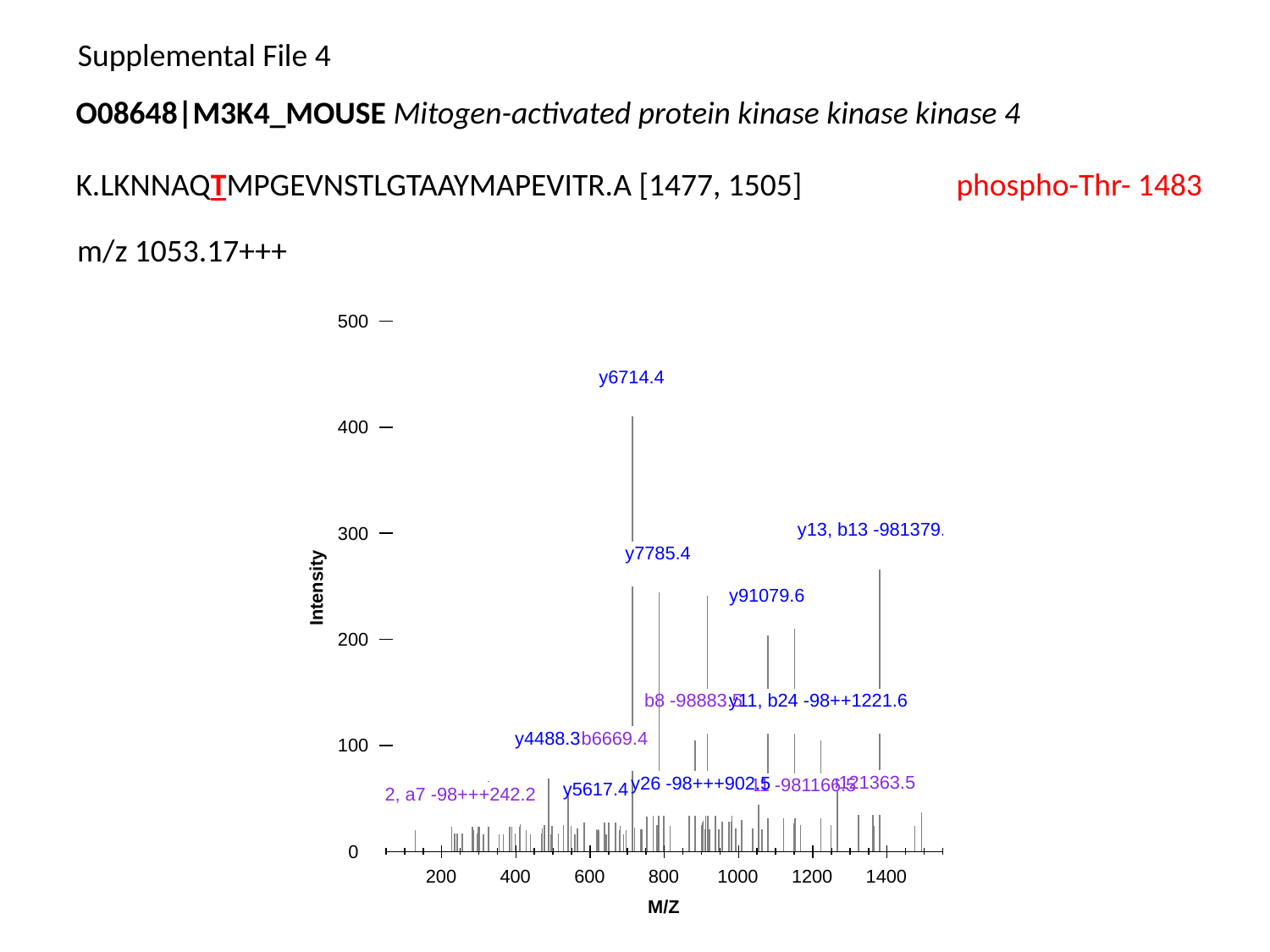

Supplemental File 4
O08648|M3K4_MOUSE Mitogen-activated protein kinase kinase kinase 4
K.LKNNAQTMPGEVNSTLGTAAYMAPEVITR.A [1477, 1505]
phospho-Thr- 1483
m/z 1053.17+++

## Slide 5
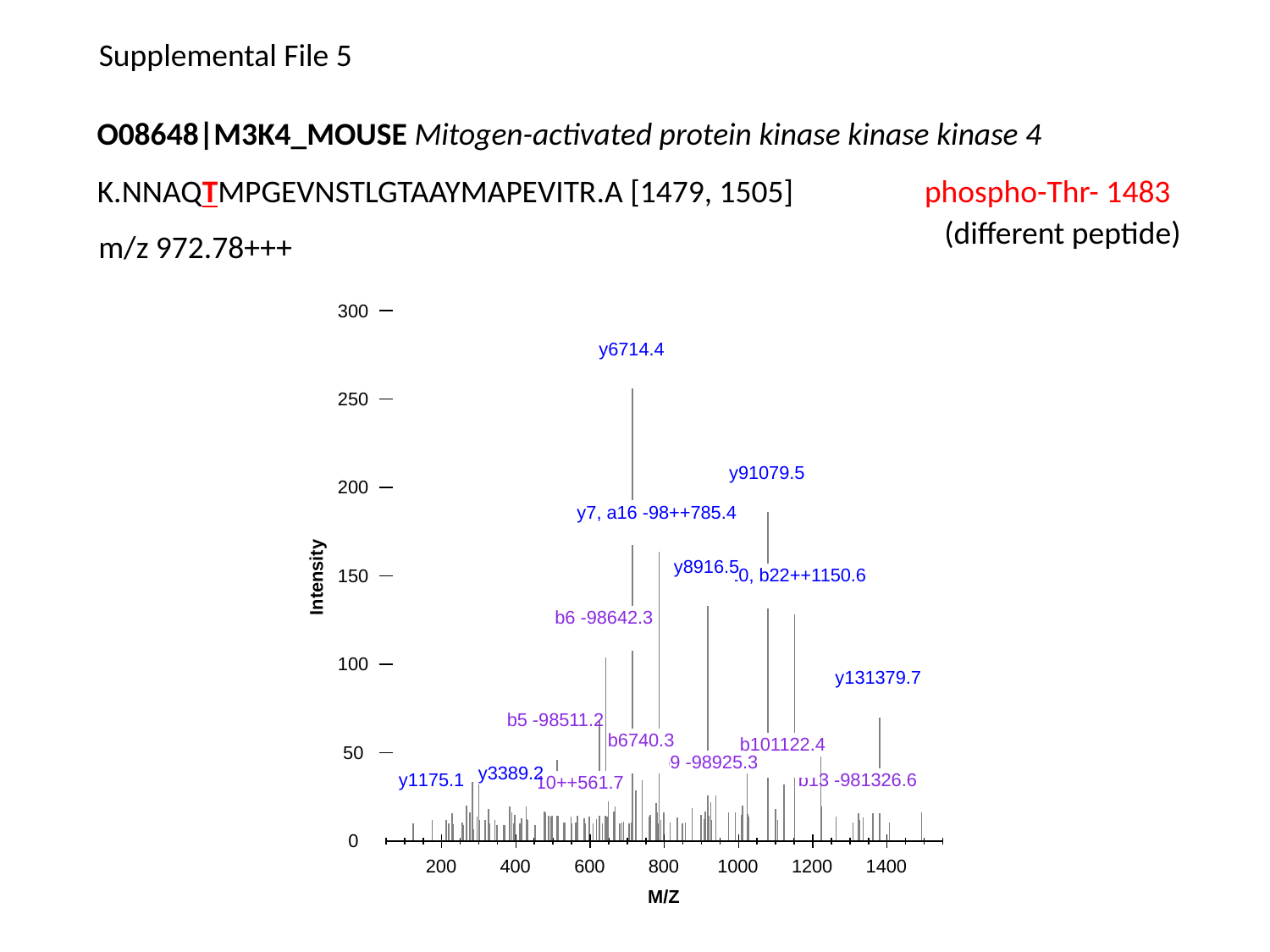

Supplemental File 5
O08648|M3K4_MOUSE Mitogen-activated protein kinase kinase kinase 4
K.NNAQTMPGEVNSTLGTAAYMAPEVITR.A [1479, 1505]
phospho-Thr- 1483
(different peptide)
m/z 972.78+++
